# A meta‐analysis of functional magnetic resonance imaging studies of eye movements and visual word reading

**DOI:** 10.1002/brb3.683

**Published:** 2017-04-04

**Authors:** Wei Zhou, Hua Shu

**Affiliations:** ^1^ Beijing Key Lab of Learning and CognitionDepartment of PsychologyCapital Normal UniversityBeijingChina; ^2^ Beijing Advanced Innovation Center for Imaging TechnologyCapital Normal UniversityBeijingChina; ^3^ State Key Laboratory of Cognitive Neuroscience and LearningIDG/McGovern Institute for Brain ResearchBeijing Normal UniversityBeijingChina

**Keywords:** functional magnetic resonance imaging, meta‐analysis, prosaccade, visual word reading

## Abstract

**Introduction:**

The pattern of eye movements during reading is substantially correlated with linguistic factors. While there have been a large number of studies on the neural mechanisms of eye movements and word reading separately, a limited number of studies have compared the activation patterns of these two processes and discussed the associations of their corresponding brain regions within the framework of naturalistic reading.

**Methods:**

This study conducted a meta‐analysis of the existing functional magnetic resonance imaging literature on prosaccades and visual word reading using the activation likelihood estimation algorithm.

**Results:**

Our main finding was that, although prosaccades and word reading mainly activated dorsal and ventral brain areas, respectively, they both activated the left precentral gyrus (PreCG), left superior parietal lobe, right PreCG, right lingual gyrus, and bilateral medial frontal gyrus.

**Conclusion:**

These findings provide new insights into cognitive processes involved with naturalistic reading, which requires both eye movements and word reading.

## 1 Introduction

Naturalistic reading requires precise integration of vision, attention, and linguistic processing. In previous studies of reading, typically, single words have been presented to readers one by one with various associated tasks, such as lexical decision making, semantic categorization, or covert or overt naming (for a review, see Price, 2012). This serial visual presentation paradigm is frequently used in most functional magnetic resonance imaging (fMRI) studies of reading, including both word reading (e.g., Mechelli, Friston, & Price, 2000) and sentence reading (e.g., Pallier, Devauchelle, & Dehaene, 2011). However, there is a salient difference between reading words one by one and reading an entire sentence at once. That is, naturalistic sentence reading requires visual attention to direct a series of eye movements through the text. Much effort has been made to examine the neural mechanisms of word reading (for a review, see Price, 2012) and eye movements (for reviews, see Munoz & Everling, 2004; Pierrot‐Deseilligny, Milea, & Muri, 2004) separately. Recent studies have attempted to explore the neural mechanisms of reading with eye movements (e.g., Choi, Desai, & Henderson, 2014; Hillen et al., 2013; Richlan et al., 2014). However, the common and unique neural substrates of these two processes are still unknown. Hence, there is a need for a meta‐analytic approach to identify common and distinct networks involved in word reading and saccade tasks.

Word reading, a simplified reading task, requires readers to view isolated words or characters with minimal eye movements. In this way, researchers can focus on the processes of orthography, phonology, and semantics. Consequently, prior research has mainly found the involvement of ventral brain areas in reading, such as the left inferior frontal gyrus (IFG), left supramarginal gyrus (SMG), left angular gyrus, and left ventral occipitotemporal cortex (VOT; Cattinelli, Borghese, Gallucci, & Paulesu, 2013; Price, 2012; Pugh et al., 2000). One notable finding is that the ventral visual stream plays a key role in visual reading. With visual word recognition tasks, researchers have consistently found that the ventral visual stream is involved in extracting the visual orthographic information of printed words (for a review, see Dehaene & Cohen, 2011). However, relatively less attention has been paid to the role of the dorsal attention‐related regions in reading. Recently, some studies have underscored that the dorsal attention‐related regions (e.g., the intraparietal sulcus [IPS] and superior parietal lobe [SPL]) may contribute to the processing of single characters/word reading, especially for degraded/distorted words (e.g., Cohen, Dehaene, Vinckier, Jobert, & Montavont, 2008) or stimuli with complex orthography (e.g., Xu, Wang, Chen, Fox, & Tan, 2015).

In contrast to word reading, typical eye‐movement tasks require participants to move their eyes between multiple stimuli/positions (for reviews, see Rayner, 1998, 2009). To focus on visual attention factors, classical eye‐movement paradigms usually utilize simple visual stimuli. For instance, subjects make visually guided saccades from a central fixation point toward a peripheral target, such as a dot or a geometric shape, in each trial of the prosaccade task (Hallett, 1978; Hutton, 2010), which is a popular paradigm to explore the neural and cognitive mechanisms of eye movements. Previous neuroimaging studies have shown that frontoparietal attentional regions play a critical role in eye movements (Corbetta & Shulman, 2002; Simon, Mangin, Cohen, Le Bihan, & Dehaene, 2002), consistent with their function for spatial representation and spatial updating (Merriam, Genovese, & Colby, 2003; Pertzov, Avidan, & Zohary, 2011; Silver & Kastner, 2009). In a recent meta‐analysis, Jamadar, Fielding, and Egan (2013) have used the activation likelihood estimation method (ALE; Turkeltaub et al., 2012; Eickhoff et al., 2009; Eickhoff, Bzdok, Laird, Kurth, & Fox, 2012) to compare the neural networks of prosaccades and antisaccades. At the cortical level, they found that the network of prosaccades includes the primary visual cortex, extrastriate cortex, parietal eye field (PEF, in the posterior parietal cortex), frontal eye field (FEF, in the superior part of the prefrontal gyrus), and supplementary eye field (SEF, in the medial frontal gyrus [MedFG]).

As noted above, word reading and eye movements are both essential to naturalistic reading. However, neither of these tasks alone can summarize the features of naturalistic reading. While isolated word reading does not require overt eye movements, traditional eye‐movement paradigms only use very simple stimuli for saccade‐targeting. These two processes are complementary and should be interactive in naturalistic reading. Therefore, two issues concerning word reading and eye movements at the cortical level should be clarified. First, what is the distinction in the functional topography of the brain between these two processes? Although prior research suggests that word reading and eye movements mainly recruit ventral and dorsal brain regions, respectively, both tasks have been reported to activate similar regions in the frontal, parietal, and occipitotemporal cortices. Hence, a further examination of the inconsistent subregions used for these separate processes is needed. Second, are there any commonly used brain regions for these two processes? As both tasks contain visual and attentional components, we expect that there are shared brain regions for these common cognitive components. The commonly used brain regions are potentially important in naturalistic reading. Our previous research using resting‐state fMRI (Zhou, Xia, Bi, & Shu, 2015) proposed that the middle frontal gyrus (MFG), an overlapping part of the eye‐movement network and word‐reading network, plays a modulatory role in naturalistic reading. A meta‐analysis based on task‐driven fMRI studies may highlight more mutually used regions for word reading and eye movements.

To address these questions, thist study conducted a meta‐analysis of the existing fMRI literature on prosaccades and visual word reading tasks. We selected these two basic paradigms of eye movements and word reading to exclude higher‐level cognitive factors such as memory or semantic processes. The goals of our investigation were twofold. First, we aimed to examine the distinction in brain activation between prosaccades and word reading. The inconsistent regions will help researchers to identify and distinguish between the brain activation of prosaccades and word reading in future studies, especially for tasks such as naturalistic reading, which includes both processes. Second, it will also be helpful to discover the consistency of brain involvement in prosaccades and word reading. The overlapping regions could underlie common cognitive factors of the modulation of prosaccades and word reading.

## 2 Materials and Methods

### 2.1 The tasks of interest

In the prosaccade paradigm, participants were required to perform simple saccadic eye movements toward a peripheral target when it appeared randomly in the right or left visual field (for a review, see Hutton & Ettinger, 2006). In visual word reading, participants were required to passively see the word stimuli or make orthography judgement tasks (for a review, see McCandliss, Cohen, & Dehaene, 2003).

### 2.2 Stimuli and procedure

A systematic search strategy was used to identify relevant studies. First, we used the coordinate database (Fox & Lancaster, 2002; Fox et al., 2005; Laird et al., 2005) in Brainmap Sleuth (http://brainmap.org/sleuth/index.html; RRID:SCR_002555) because it contains neuroimaging coordinates classified as saccade and word reading tasks. The terms “[Image Modality = fMRI] AND [Paradigm = Saccade]” were entered to search for studies of eye movements; the terms “[Image Modality = fMRI] AND [Behavioral Domain = Cognition.Language‐Orthography]” were entered to search for studies of word reading. At the same time, we conducted a PubMed search (https://www.ncbi.nlm.nih.gov/pubmed) using the search terms “prosaccade” and “fMRI” for studies of eye movements, and “reading,” “orthography,” and “fMRI” for studies of word reading.

Out of the 73 articles identified as studies of eye movements, 19 studies fulfilling the following criteria were included in the meta‐analysis (Table 1): (1) used prosaccade tasks other than saccades in darkness, anti‐saccades, memory‐guided saccades, successive saccades, or saccades in smooth pursuit; (2) used healthy adults as participants and not children or psychiatrically/neurologically impaired subjects; (3) used the central fixation as the baseline and not high‐level baselines; and (4) used whole‐brain scanning and reported complete coordinates of activation in standardized stereotaxic space. Out of the 154 articles identified as studies of word reading, 18 studies fulfilling the following criteria were included in the meta‐analysis (Table 2): (1) used an isolated visual word or character for each presentation; (2) used healthy adults as participants and not children or psychiatrically/neurologically impaired subjects; (3) did not use active and overt phonology, semantic, emotional, or memory tasks; (4) used central fixation, rest or simple visual stimuli as the baseline, and not complex linguistic stimuli; and (5) used whole‐brain scanning and reported the complete coordinates of activation in standardized stereotaxic space. Finally, we identified 19 papers, 335 subjects, 23 contrasts, and 344 locations of foci for the meta‐analysis of eye movements and 18 papers, 364 subjects, 26 contrasts, and 428 locations of foci for the meta‐analysis of word reading.

**Table 1:** Studies of eye movements included in the meta‐analysis

Author (year)	Contrasts	Stimuli	*N*	No. of foci
Bär, Hauf, Barton, and Abegg (2016)	Prosaccade > fixation	Circle	14	13
Herweg et al. (2014)	Prosaccade > fixation	Dot	26	12
Lukasova et al. (2014)	Prosaccade > fixation	Dot	15	15
	Prosaccade > fixation	Dot	15	10
	Prosaccade > fixation	Dot	15	12
	Prosaccade > fixation	Dot	15	12
Aichert, Williams, Möller, Kumari, and Ettinger (2012)	Prosaccade > fixation	Dot	54	18
Nelles, Greiff, Pscherer, and Esser (2009)	Prosaccade > fixation	Dot	11	9
van Broekhoven et al. (2009)	Prosaccade > fixation	Dot	17	22
Postle and Hamidi (2007)	Prosaccade > fixation	Circle	12	42
Brown, Goltz, Vilis, Ford, and Everling (2006)	Prosaccade > fixation	Dot	10	17
	Prosaccade > fixation	Dot	10	10
Matsuda et al. (2004)	Prosaccade > fixation	Geometrical	21	9
Astafiev et al. (2003)	Prosaccade > fixation	Asterisk	15	10
Simon et al. (2002)	Prosaccade > fixation	Square	10	17
Gitelman, Parrish, Friston, and Mesulam (2002)	Prosaccade > central	Digits	17	17
Gagnon, O'Driscoll, Petrides, and Pike (2002)	Prosaccade > fixation	Square	7	10
Heide et al. (2001)	Prosaccade > fixation	Geometrical	6	10
Kimmig et al. (2001)	Prosaccade > fixation	Asterisk	15	14
Connolly, Goodale, Desouza, Menon, and Vilis (2000)	Prosaccade > fixation	Geometrical	7	7
Perry and Zeki (2000)	Prosaccade > fixation	Circle or triangle	7	17
Corbetta et al. (1998)	Prosaccade > fixation	Asterisk	6	29
Luna et al. (1998)	Prosaccade > fixation	Circle	10	12

**Table 2:** Studies of word reading included in the meta‐analysis

Author (year)	Contrasts	Tasks	*N*	No. of foci
Wang et al. (2015)	Real/pseudo characters > rest	Lexical decision	16	10
	Real/pseudo words > rest	Lexical decision	16	13
Zhang, Xiao, and Weng (2012)	Word > rest	Lexical decision	28	43
Liu et al. (2008)	Real characters > checkerboard	Font size judgement	14	5
	Pseudo characters > checkerboard	Font size judgement	14	10
Liu, Dunlap, Fiez, and Perfetti (2007)	Real words > fixation	Covert reading	23	14
	Pseudo words > fixation	Covert reading	23	14
Meschyan and Hernandez (2006)	Words > rest	Covert reading	12	12
Bonner‐Jackson, Haut, Csernansky, and Barch (2005)	Words > fixation	Letter discriminate	26	50
Ragland et al. (2005)	Words > fixation	Uppercase judgement	14	10
Booth et al. (2004)	Words > rest	Visual discrimination	16	9
Eyler, Olsen, Jeste, and Brown (2004)	Letter strings > fixation	Letter detection	10	4
Cohen et al. (2003)	(words + letter strings) > fixation	Covert reading	9	11
	(words + letter strings) > checkerboard	Covert reading	9	7
Ding et al. (2003)	Characters > fixation	Radical judgement	6	9
Longcamp, Anton, Roth, and Velay (2003)	Letter > line	Passive viewing	11	7
Kubicki et al. (2003)	Words > rest	Uppercase judgement	9	3
Fu, Chen, Smith, Iversen, and Matthews (2002)	Characters (present quickly) > fixation	Covert reading	8	35
	Characters (present slowly) > fixation	Covert reading	8	20
Dehaene et al. (2001)	Words > rest	Covert reading	37	15
Mechelli et al. (2000)	Words > rest	Covert reading	6	18
	Pseudo words > rest	Covert reading	6	20
Stevens, Skudlarski, Gatenby, and Gore (2000)	Letter strings > rest	Letter detection	10	20
Tagamets, Novick, Chalmers, and Friedman (2000)	Words > shapes	Visual discrimination	11	18
	Pseudowords > shapes	Visual discrimination	11	20
	Letter strings > shapes	Visual discrimination	11	31

### 2.3 Data analyses

#### 2.3.1 Creation of ALE maps

The meta‐analysis was performed using the ALE algorithm (Eickhoff et al., 2009, 2012; Turkeltaub et al., 2012) found in the GingerALE2.3 software (http://brainmap.org/ale/; RRID:SCR_014921). In the ALE approach, spatial probability distributions for the foci were modeled at the center of three‐dimensional Gaussian functions and the Gaussian distributions were aggregated across the entire set of experiments to generate a map of consistencies among studies that estimated the likelihood of activation for each voxel—the ALE statistic (Eickhoff et al., 2009). Coordinates reported in the Talairach space were first transformed into the Montreal Neurological Institute brain template using the appropriate transformation algorithms implemented in GingerALE.

#### 2.3.2 Contrast and conjunction analyses

To evaluate differences and similarities in brain activation between eye movements and word reading, the software conducted a contrast analysis to compare the two ALE datasets and a conjunction analysis using the voxel‐wise minimum value of the input ALE images (Eickhoff, Bzdok, Laird, Roski, & Caspers, 2011). After 5,000 permutations, we had a voxel‐wise *p*‐value image showing where the true data values sit on the distribution of values in that voxel. The FDR method was used to correct for multiple comparisons at a significance threshold of *p *<* *.05 and a cluster threshold of 200 mm^3^.

## 3 Results

### 3.1 Regions for prosaccades

**Figure 1:**
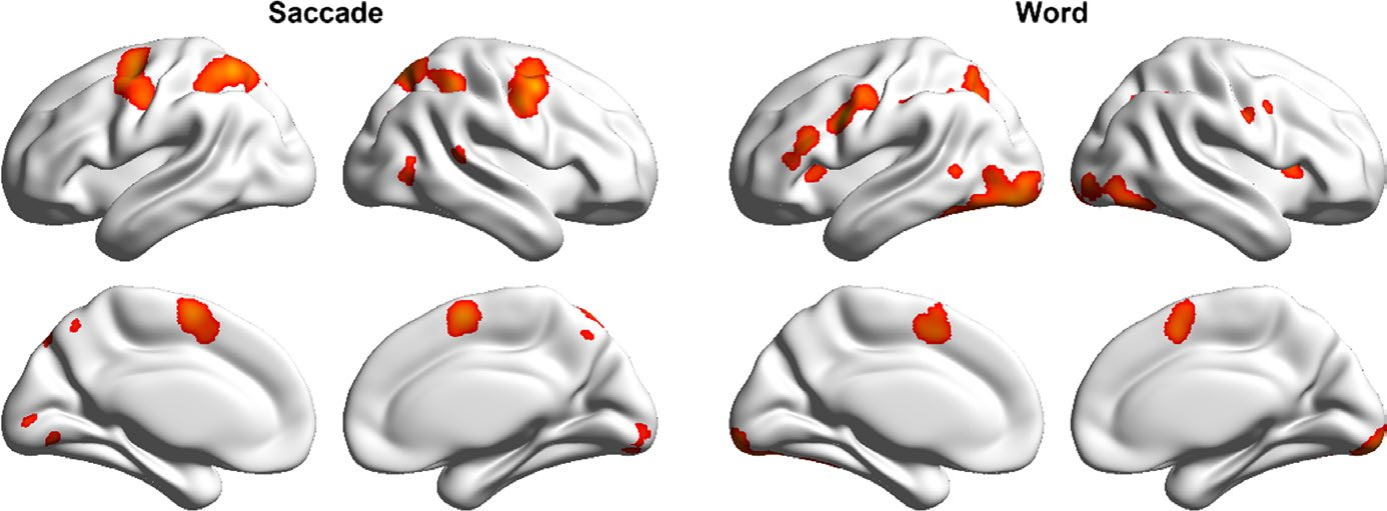
Activation likelihood maps for the saccadic (left panel) and word reading tasks (right panel)

The areas commonly activated in saccadic tasks across all studies are presented in Table 3 and Figure 1. These activations were largely bilateral and included the superior part of the precentral gyrus (PreCG), MedFG, SPL, precuneus (PreCUN), occipital gyrus (OG), putamen (PUT), right superior temporal sulcus (STS), and left cerebellum. These regions were mainly located in the dorsal attention stream and visual association cortex.

**Table 3:** Montreal Neurological Institute (MNI) coordinates, volume (mm; each voxel is equivalent to 8 mm^3^), activation likelihood estimation (ALE) values, and brain regions for prosaccades and word reading, respectively

Cluster no.	Volume (mm^3^)	ALE	MNI			Regions
			*x*	*y*	*z*	
Saccade						
1	9,312	0.039	−33	−5	52	Left precentral gyrus
2	8,952	0.033	39	−2	50	Right precentral gyrus
3	7,680	0.034	−30	−55	55	Left superior parietal lobe
4	6,160	0.027	27	−59	55	Right superior parietal lobe
5	5,912	0.042	0	2	57	Medial frontal gyrus
6	1,680	0.023	15	−89	−6	Right lingual gyrus
7	1,392	0.021	−22	6	3	Left putamen
8	1,184	0.029	21	5	5	Right putamen
9	824	0.015	−9	−72	−11	Left cerebellum
10	752	0.017	−23	−74	25	Left superior occipital gyrus
11	560	0.019	60	−41	10	Right superior temporal sulcus
12	552	0.015	46	−66	4	Right middle temporal gyrus
13	456	0.016	−14	−79	48	Left superior parietal lobe
14	408	0.014	30	−73	27	Right middle occipital gyrus
15	376	0.013	−12	−88	5	Left calcarine
16	256	0.013	−1	−83	−13	Left calcarine
17	240	0.016	−37	−66	−22	Left cerebellum
18	200	0.012	3	−66	48	Right precuneus
19	200	0.013	−5	−62	54	Left precuneus
Word						
1	14,784	0.032	−34	−79	−12	Left lingual gyrus
2	10,784	0.028	30	−81	−12	Right lingual gyrus
3	4,384	0.026	0	7	53	Medial frontal gyrus
4	4,360	0.042	−48	2	35	Left precentral gyrus
5	3,824	0.023	−27	−62	46	Left superior parietal lobe
6	2,424	0.023	31	−59	39	Right inferior parietal lobe
7	1,744	0.024	−48	27	18	Left inferior frontal gyrus
8	672	0.016	32	14	5	Right insula
9	552	0.016	−31	19	4	Left insula
10	448	0.017	−29	−74	28	Left superior occipital lobe
11	392	0.017	−59	−56	5	Left middle temporal gyrus
12	320	0.015	53	−8	33	Right precentral gyrus
13	320	0.014	−51	−32	40	Left inferior parietal lobe
14	272	0.014	14	−8	6	Right thalamus
15	256	0.015	46	1	37	Right precentral gyrus

### 3.2 Regions for word reading

Regions consistently activated in word reading tasks are presented in Table 3 and Figure 1. These activations mainly included the bilateral PreCG, MedFG, inferior parietal lobe (IPL), OG, insula (INS), and left IFG, SPL, and MTG.

### 3.3 Unique regions

Uniquely activated regions in prosaccades and word reading tasks are presented in Table 4. The uniquely activated regions for prosaccades were mainly in dorsal visual regions, including the bilateral PreCG (the superior part), SPL, MedFG, PreCUN, calcarine, and left cerebellum. The uniquely activated regions for word reading were mainly situated in ventral regions, including the bilateral fusiform (FFG), left PreCG (the inferior part), and IFG.

**Table 4:** Montreal Neurological Institute (MNI) coordinates, volume (mm^3^; each voxel is equivalent to 8 mm^3^), and uniquely activated brain regions for saccadic and word reading tasks

Cluster no.	Volume (mm^3^)	MNI			Regions
		*x*	*y*	*z*	
Saccade > word					
1	4,768	−34	−5	50	Left precentral gyrus
2	4,528	40	−1	50	Right precentral gyrus
3	2,520	−32	−51	58	Left superior parietal lobe
4	2,456	19	−67	59	Right superior parietal lobe
5	1,320	−1	−3	63	Left medial frontal gyrus
6	664	−10	−72	−10	Left cerebellum
7	232	−14	−79	49	Left superior parietal lobe
8	200	3	−66	48	Right precuneus
9	200	−4	−62	54	Left precuneus
Word > saccade					
1	7,072	−37	−73	−13	Left fusiform
2	2,024	−45	6	30	Left precentral gyrus
3	624	−51	28	16	Left inferior frontal gyrus
4	216	41	−63	−15	Right fusiform

### 3.4 Commonly activated regions

**Figure 2:**
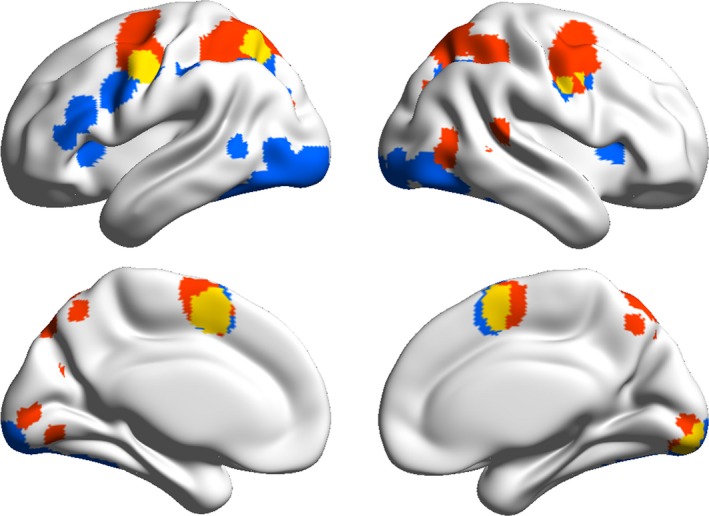
Overlaid activation likelihood maps for prosaccades and word reading. Red: saccade; Blue: word; Yellow: overlaid

Commonly activated regions for prosaccades and word reading were calculated using conjunction analysis (see Table 5 for results). For illustration, Figure 2 presents the overlaid activation map between the two tasks. There were five identified brain regions including the left PreCG (the middle part), left SPL, right PreCG (only in overlaid activation map), right lingual gyrus (LING), and bilateral MedFG.

**Table 5:** Montreal Neurological Institute (MNI) coordinates, volume (mm^3^; each voxel is equivalent to 8 mm^3^), and commonly activated brain regions for prosaccades and word reading

Cluster no	Volume (mm^3^)	MNI			Regions
		*x*	*y*	*z*	
1	2,960	−1	5	54	Medial frontal gyrus
2	1,208	16	−90	−6	Right lingual gyrus
3	1,040	−25	−63	53	Left superior parietal lobe
4	736	−47	−7	43	Left precentral gyrus

## 4 Discussion

Motivated by a concern regarding the neural association of eye movements and word reading processes, both of which are important factors for naturalistic reading, this study compared the activation networks of prosaccades and visual word reading. To the best of our knowledge, this is the first meta‐analysis study including these two processes simultaneously. We have shown that prosaccade‐related regions are mainly located in the dorsal visual stream, whereas word reading‐related activations are mainly located in ventral brain regions. The common regions for these two processes included the left PreCG, left SPL, right PreCG, right LING, and bilateral MedFG. We propose that the neural mechanisms of these two processes can be discussed in the framework of naturalistic reading.

### 4.1 Functional dissociations: dual‐visual routes and subregions

While previous studies of reading tended to focus on the role of ventral brain regions, this study attempted to emphasize the involvement of dorsal visual regions. According to the dual‐route theory of visual processing (e.g., Goodale & Milner, 1992), the division of labor between a dorsal “where” stream and a ventral “what” stream is one of the most fundamental principles of information processing in the brain (Ungerleider & Haxby, 1994). Similarly, processes involved in naturalistic text reading may also follow this dual‐route principle. The current results clearly illustrate a pattern of dual‐visual routes for two processes that are relevant to naturalistic reading: prosaccades mainly activated dorsal visual regions, whereas visual word reading mainly activated ventral visual regions. Consistently, a meta‐analysis of eye movements by Jamadar et al. (2013) reported the activation of bilateral FEF/PreCG, PEF/SPL, SEF/MedFG, and left LING in the prosaccade task. In addition, a meta‐analysis of Chinese orthographic processing by Wu, Ho, and Chen (2012) identified the left PreCG, SPL, VOT, PreCUN, cuneus, cingulate gyrus, and right PreCUN. By including studies of visual word reading across alphabetic and logographic writing systems, we observed a similar pattern of activation to that found by Wu et al. (2012), but additionally identified the bilateral MedFG, IPL, INS, left IFG, and PUT.

There has been a large body of research using meta‐analytical approaches in the field of single‐word recognition (Jobard, Crivello, & Tzourio‐Mazoyer, 2003; Martin, Schurz, Kronbichler, & Richlan, 2015; Taylor, Rastle, & Davis, 2013; Turkeltaub, Eden, Jones, & Zeffiro, 2002). In general, our results were in agreement with previous findings that ventral regions, such as the VOT, MTG, and IFG, are activated during single word processing. It is noted that these meta‐analysis studies included orthographic, phonological, and semantic tasks for single word recognition and contained both silent and oral reading. Consequently, researchers have also found “dual routes” for single word reading (e.g., Coltheart, Rastle, Perry, Langdon, & Ziegler, 2001; Jobard et al., 2003; Taylor et al., 2013). This dual‐route system is comprised of a dorsal phonological pathway (i.e., the STG, SMG, and opercular part of the IFG) and a ventral lexical‐semantic pathway (i.e., the VOT, MTG, and triangular part of the IFG) among left perisylvian regions. As we mainly focused on the orthographic aspect of lexical processing in the silent reading, activation of phonological regions, such as the STG and SMG, were not observed in this study. However, the dorsal phonological route and dorsal visual route should be distinguished, and the relationships between these two dorsal routes require further investigation.

Although previous studies on meta‐analyses have demonstrated a general functional dissociation of the dorsal and ventral visual regions for eye movements and word reading, respectively, the function of some regions is still unclear. For example, the SPL/IPS has been reported to be activated in both eye movements and language‐related tasks (e.g., Simon et al., 2002). The cause of activation in the SPL/IPS during naturalistic reading cannot be readily inferred by literature reviews or separate meta‐analyses for these two tasks. In such cases, subregions of the regions revealed in the current study will help researchers to identify and distinguish the activation of prosaccades and word reading in future studies of naturalistic reading.

We have found that prosaccades recruited more superior parts of the frontoparietal cortex, more posterior parts of the medial frontal cortex, and fewer occipital/temporal regions relative to word reading. These findings are basically consistent with the known organization of brain function. The superior part of the PreCG, the FEF, is related to goal‐directed saccades and spatial processing (Corbetta & Shulman, 2002), whereas, the inferior part of the PreCG, the IFG, is related to the identification of objects such as words and faces (McDermott, Buckner, Petersen, Kelley, & Sanders, 1999). While the SPL is related to spatial processing, such as the formation of spatial maps for the control of eye movements (Graziano & Gross, 1998), the IPL serves as an orthography‐phonology transmitter in the word reading and auditory‐motor interface in language processing (Hickok & Poeppel, 2000). Whereas, the posterior part of the MedFG/supplementary motor area (SMA) is more closely tied to motor output, the anterior part of the MedFG/SMA could be involved in higher level cognitive processes (Alario, Chainay, Lehericy, & Cohen, 2006). As the primary visual cortex mainly processes simple visual stimuli, such as dots or geometric drawings, more extensive regions in the occipitotemporal cortex provide a neural basis for visual word‐form detection (Vinckier et al., 2007). In summary, there is a hierarchical distribution and organization of brain regions for prosaccades and word reading. The coordinates of the subregions for these two tasks can be used as regions of interest in future data analyses.

### 4.2 Functional integration: common cognitive factors and the potential role of coordination for commonly activated regions

A novel finding of this study is that brain regions in the PreCG, MedFG, parietal lobe, and occipital gyrus are activated during both prosaccades and visual word reading. A direct explanation of the mutually activated regions is that they serve as common cognitive factors of prosaccades and word reading. When researchers investigate the neural mechanisms of reading with saccades, these mutual brain regions deserve special attention. On one hand, it might provide a confounding factor when distinguishing the activation for eye movements and word reading in these regions during naturalistic reading. On the other hand, these regions are situated in the transitive borders between distinctive networks for eye movements and word reading and are likely to engage in interactions between eye movements and word reading in naturalistic reading. In other words, the mutually activated regions might serve in the coordination of eye movements and word reading.

The function of the commonly activated brain regions in this study can characterize most of the common cognitive factors between prosaccades and word reading. Because both tasks start with vision and require visual attention, the overlapping regions are generally related to those functions. Previous research has indicated that the MFG is a transition region between the FEF and IFG (Courtney, Petit, Maisog, Ungerleider, & Haxby, 1998), and is recruited for visuospatial manipulation in both visual word recognition (Tan, Spinks, Eden, Perfetti, & Siok, 2005; Wu et al., 2012) and spatial processing tasks (Belger et al., 1998; Carlson et al., 1998; McCarthy et al., 1996). The SPL/IPS may serve a role in spatial relationship analysis for both saccade‐targeting and processing of sequentially arranged letters in a word (Simon et al., 2002). While the MedFG/SMA is related to the preparation of movement and the control of sequences of movement (Russo & Bruce, 2000), it has also been found to be involved in lexical selection, linear sequence encoding, and control of motor output for word production (Alario et al., 2006). The commonly activated region in the occipital cortex for prosaccades and word reading is the LING, which is associated with basic visual processing.

Interestingly, an increasing number studies using resting‐state fMRI have demonstrated that these overlapping brain regions are functionally connected to regions involved in reading and visual attention (Koyama et al., 2010; Vogel, Miezin, Petersen, & Schlaggar, 2012; Zhou et al., 2015). As reported by Zhou et al. (2015), the middle part of the prefrontal gyrus, the MFG, is functionally connected to seeds of the IPS and visual word form area (VWFA), which were selected on the basis of eye movement and word reading research, respectively. They found that the strengths of these functional connections were positively correlated with the naturalistic reading score but not with the word reading score, suggesting that the MFG is crucial in naturalistic reading. Moreover, Zhou et al. (2016) found that there was a top‐down effect from the MFG to both the IPS and VWFA during naturalistic text reading. As a result, we believe that the middle part of the prefrontal/PreCG plays a role in the integration and modulation of eye movements and word reading during naturalistic reading. Likewise, the SPL/IPS may play a role in the perceptual‐motor transition, and the SMA may coordinate the planning of eye movements and word reading during naturalistic reading. Taken together, we propose that the mutually activated areas of these two cognitive systems could act as a hub to connect distributed systems in a complex task such as naturalistic reading. However, these propositions require further investigation.

### 4.3 Neural mechanisms of reading with eye movements

This study attempted to examine the neural mechanisms of reading with eye movements using a meta‐analytical approach. The results will facilitate our understanding of the relationship between brain areas for word reading and eye movements. In a real‐world context, however, reading and eye movements occur concurrently with another. The relationship between word reading and eye movements should be studied in an ecological context. More recently, there have been interesting developments using self‐paced reading tasks in fMRI experiments with eye‐movement recording (Henderson, Choi, Luke, & Desai, 2015; Schuster, Hawelka, Hutzler, Kronbichler, & Richlan, 2016; Schuster, Hawelka, Richlan, Ludersdorfer, & Hutzler, 2015). These studies have observed task‐dependent brain activation for reading‐related regions (Schuster et al., 2015) and have provided evidence that fixation duration was associated with activation in oculomotor and language areas during text reading (Henderson et al., 2015). Interestingly, Schuster et al. (2016) found higher activation within precentral, superior parietal, and occipital regions (including the LING) when an upcoming word was about to be skipped as compared with when it was to be fixated. This pattern of results resembles the presently observed overlapping regions between visual word recognition and eye movement behavior. The results of this study may help to interpret why skipping activates those specific regions during naturalistic reading. Skipping, a phenomenon that can only happen during reading with eye movements, requires a relatively intensive coordination between the processing of the parafoveal word and the planning of the next saccade. As a result, it relies more on the mutually required regions for these two processes.

## 5 Conclusion

In conclusion, our results indicate that, although prosaccades and word reading mainly activate the dorsal and ventral brain areas, respectively, they both activate the left PreCG, left SPL, right PreCG, right LING, and bilateral MedFG. These findings suggest that while prosaccades and word reading recruit separate networks, naturalistic reading requires the cooperation of dorsal‐ventral networks, which may be coordinated by regions mutually activated by prosaccades and word reading. Thus, this study has provided new insights into the cognitive processes involved in naturalistic reading, which requires both eye movement and word reading processes. The limitation of this study is that only studies using very simple eye movement and word reading tasks were included in the meta‐analysis. Future efforts should be directed to closer scrutinize the function and association of these mutually required regions in comprehensive naturalistic/saccadic reading tasks, especially making use of the initial findings of the present study.

## Conflict of Interest

None declared.
